# The effectiveness of art therapy for anxiety in adults: A systematic review of randomised and non-randomised controlled trials

**DOI:** 10.1371/journal.pone.0208716

**Published:** 2018-12-17

**Authors:** Annemarie Abbing, Anne Ponstein, Susan van Hooren, Leo de Sonneville, Hanna Swaab, Erik Baars

**Affiliations:** 1 Faculty of Health, University of Applied Sciences Leiden, Leiden, The Netherlands; 2 Clinical Neurodevelopmental Sciences, Faculty of Social Sciences, Leiden University, Leiden, The Netherlands; 3 KenVak, Research Centre for the Arts Therapies, Heerlen, The Netherlands; 4 Centre for the Arts Therapies, Zuyd University of Applied Sciences, Heerlen, The Netherlands; 5 Faculty of Psychology and Educational Sciences, Open University, Heerlen, The Netherlands; NIH/NCI/DCP/BRG, UNITED STATES

## Abstract

**Background:**

Anxiety disorders are one of the most diagnosed mental health disorders. Common treatment consists of cognitive behavioral therapy and pharmacotherapy. In clinical practice, also art therapy is additionally provided to patients with anxiety (disorders), among others because treatment as usual is not sufficiently effective for a large group of patients. There is no clarity on the effectiveness of art therapy (AT) on the reduction of anxiety symptoms in adults and there is no overview of the intervention characteristics and working mechanisms.

**Methods:**

A systematic review of (non-)randomised controlled trials on AT for anxiety in adults to evaluate the effects on anxiety symptom severity and to explore intervention characteristics, benefitting populations and working mechanisms. Thirteen databases and two journals were searched for the period 1997 –October 2017. The study was registered at PROSPERO (CRD42017080733) and performed according to the Cochrane recommendations. PRISMA Guidelines were used for reporting.

**Results:**

Only three publications out of 776 hits from the search fulfilled the inclusion criteria: three RCTs with 162 patients in total. All studies have a high risk of bias. Study populations were: students with PTSD symptoms, students with exam anxiety and prisoners with prelease anxiety. Visual art techniques varied: trauma-related mandala design, collage making, free painting, clay work, still life drawing and house-tree-person drawing. There is some evidence of effectiveness of AT for pre-exam anxiety in undergraduate students. AT is possibly effective in reducing pre-release anxiety in prisoners. The AT characteristics varied and narrative synthesis led to hypothesized working mechanisms of AT: induce relaxation; gain access to unconscious traumatic memories, thereby creating possibilities to investigate cognitions; and improve emotion regulation.

**Conclusions:**

Effectiveness of AT on anxiety has hardly been studied, so no strong conclusions can be drawn. This emphasizes the need for high quality trials studying the effectiveness of AT on anxiety.

## Introduction

Anxiety disorders are disorders with an ‘abnormal’ experience of fear, which gives rise to sustained distress and/ or obstacles in social functioning [1]. Among these disorders are panic disorder, social phobia, agoraphobia, specific phobia, obsessive-compulsive disorder (OCD) and generalized anxiety disorder (GAD). The prevalence of anxiety disorders is high: 12.0% in European adults [2] and 10.1% in the Dutch population [3]. Lifetime prevalence for women ranges from 16.3% [2, 4] to 23.4% [3] and for men from 7.8% to 15.9% [2, 3] in Europe. It is the most diagnosed mental health disorder in the US [5] and incidence levels have increased over the last half of the 20^th^ century [6].

Anxiety disorders rank high in the list of burden of diseases. According to the Global Burden of Disease study [7], anxiety disorders are the sixth leading cause of disability, in terms of years lived with disability (YLDs), in low-, middle- and high-income countries in 2010. They lead to reduced quality of life [8] and functional impairment, not only in personal life but also at work [4, 9, 10] and are associated with substantial personal and societal costs [11].

The most common treatments of anxiety disorders are cognitive behavioral therapy (CBT) and/ or pharmacotherapy with benzodiazepines, tricyclic antidepressants, monoamine oxidase inhibitors and selective serotonin reuptake inhibitors [1]. These treatments appear to be only moderately effective. Pharmacological treatment causes side effects and a significant percentage of patients (between 20–50% [12–15] is unresponsive or has a contra-indication. Combination with CBT is recommended [16] but around 50% of patients with anxiety disorders do not benefit from CBT [17].

To increase the effectiveness of treatment of anxiety disorders, additional therapies are used in clinical practice. An example is art therapy (AT), which is integrated in several mental health care programs for people with anxiety (e.g. [18, 19]) and is also provided as a stand-alone therapy. AT is considered an important supportive intervention in mental illnesses [20–22], but clarity on the effectiveness of AT is currently lacking.

AT uses fine arts as a medium, like painting, drawing, sculpting and clay modelling. The focus is on the process of creating and (associated) experiencing, aiming for facilitating the expression of memories, feelings and emotions, improvement of self-reflection and the development and practice of new coping skills [21, 23, 24].

AT is believed to support patients with anxiety in coping with their symptoms and to improve their quality of life [20]. Based on long-term experience with treatment of anxiety in practice, AT experts describe that AT can improve emotion regulation and self-structuring skills [25–27] and can increase self-awareness and reflective abilities [28, 29]. According to Haeyen, van Hooren & Hutschemakers [30], patients experience a more direct and easier access to their emotions through the art therapies, compared to verbal approaches. As a result of these experiences, AT is believed to reduce symptoms in patients with anxiety.

Although AT is often indicated in anxiety, its effectiveness has hardly been studied yet. In the last decade some systematic reviews on AT were published. These reviews covered several areas. Some of the reviews focussed on PTSD [31–34], or have a broader focus and include several (mental) health conditions [35–39]. Other reviews included AT in a broader definition of psychodynamic therapies [40] or deal with several therapies (CBTs, expressive art therapies (e.g., guided imagery and music therapy), exposure therapies (e.g., systematic desensitization) and pharmacological treatments within one treatment program) [41].

No review specifically aimed at the effectiveness of AT on anxiety or on specific anxiety disorders. For anxiety as the primary condition, thus not related to another primary disease or condition (e.g. cancer or autism), there is no clarity on the evidence nor of the employed therapeutic methods of AT for anxiety in adults. Furthermore, clearly scientifically substantiated working mechanism(s), explaining the anticipated effectiveness of the therapy, are lacking.

### Objectives

The primary objective is to examine the effectiveness of AT in reducing anxiety symptoms.

The secondary objective is to get an overview of (1) the characteristics of patient populations for which art therapy is or may be beneficial, (2) the specific form of ATs employed and (3) reported and hypothesized working mechanisms.

## Methods

### Protocol and registration

The systematic review was performed according to the recommendations of the Cochrane Collaboration for study identification, selection, data extraction, quality appraisal and analysis of the data [42]. The PRISMA Guidelines [43] were followed for reporting (S1 Checklist). The review protocol was registered at PROSPERO, number CRD42017080733 [44]. The AMSTAR 2 checklist was used to assess and improve the quality of the review [45].

### Eligibility criteria

#### Types of study designs

The review included peer reviewed published randomised controlled trials (RCTs) and non-randomised controlled trials (nRCTs) on the treatment of anxiety symptoms. nRCTs were also included because it was hypothesized that nRCTs are more executed than RCTs, for the research field of AT is still in its infancy.

Only publications in English, Dutch or German were included. These language restrictions were set because the reviewers were only fluent in these three languages.

#### Types of participants

Studies of adults (18–65 years), from any ethnicity or gender were included.

#### Types of interventions

AT provided to individuals or groups, without limitations on duration and number of sessions were included.

#### Types of comparisons

The following control groups were included: 1) inactive treatment (no treatment, waiting list, sham treatment) and 2) active treatment (standard care or any other treatment). Co-interventions were allowed, but only if the additional effect of AT on anxiety symptom severity was measured.

#### Types of outcome measures

Included were studies that had reduction of anxiety symptoms as the primary outcome measure. Excluded were studies where reduction of anxiety symptoms was assessed in non-anxiety disorders or diseases and studies where anxiety symptoms were artificially induced in healthy populations. Populations with PTSD were not excluded, since this used to be an anxiety disorder until 2013 [46].

### Searches

The following 13 databases and two journals were searched: PUBMED, Embase (Ovid), EMCare (Ovid), PsychINFO (EBSCO), The Cochrane Library (Cochrane Database of Systematic Reviews, Cochrane Central Register of Controlled Trials, Database of Abstracts of Review of Effects, Web of Science, Art Index, Central, Academic Search Premier, Merkurstab, ArtheData, Reliëf, Tijdschrift voor Vaktherapie.

A search strategy was developed using keywords (art therapy, anxiety) for the electronic databases according to their specific subject headings or structure. For each database, search terms were adapted according to the search capabilities of that database (S1 File Full list of search terms).

The search covered a period of twenty years: 1997 until October 9, 2017. The reference lists of systematic reviews—found in the search—were hand searched for supplementing titles, to ensure that all possible eligible studies would be detected.

### Study selection

A single endnote file of all references identified through the search processes was produced. Duplicates were removed.

The following selection process was independently carried out by two researchers (AA and AP). In the first phase, titles were screened for eligibility. The abstracts of the remaining entries were screened and only those that met the inclusion criteria were selected for full text appraisal. These full texts were subsequently assessed according to the eligibility criteria. Any disagreement in study selection between the two independent reviewers was resolved through discussion or by consultation of a third reviewer (EB).

### Data collection process

The data were extracted by using a data extraction spreadsheet, based on the Cochrane Collaboration Data Collection Form for intervention reviews (S1 Table Data collection form).

The form concerned the following data: aim of the study, study type, population, number of treated subjects, number of controlled subjects, AT description, duration, frequency, co-intervention(s), control description, outcome domains and outcome measures, time points, outcomes and statistics.

After separate extraction of the data, the results of the two independent assessors were compared and discussed to reach consensus.

### Risk of bias in individual studies

The risk of bias (RoB) was independently assessed by the two reviewers with the Cochrane Collaboration’s tool for assessing RoB [47]. Bias was assessed over the domains: selection bias (random sequence generation and allocation concealment), performance bias (blinding of participants and personnel), detection bias (blinding of researchers conducting outcome assessments), attrition bias (incomplete outcome data), reporting bias (selective reporting). A judgement of ‘low’, ‘high’ or ‘unclear’ risk of bias was provided for each domain. Since the RoB tool was developed for use in pharmacological studies, we followed the recommendations of Munder & Barth [48] that placed the RoB tool in the context of psychotherapy outcome research. Performance bias is defined here as "studies that did not use active control groups or did not assess patient expectancies or treatment credibility", instead of only 'blinding of participants and personnel'.

A summary assessment of RoB for each study was based on the approach of Higgins & Green [47]: overall low RoB (low risk of bias in all domains), unclear RoB (unclear RoB in at least one domain) and high RoB (unclear RoB in more than one domain or high RoB in at least one domain).

### Outcomes

The primary outcome measure was anxiety symptoms reduction (pre-post treatment). The outcomes are presented in terms of differences between intervention and control groups (e.g., risk ratios or odds ratios). Within-group outcomes are also presented, to identify promising outcomes and hypotheses for future research.

Data from studies were combined in a meta-analyses to estimate overall effect sizes, if at least two studies with comparable study populations and treatment were available that assessed the same specific outcomes. Heterogeneity was examined by calculating the I^2^ statistic and performing the Chi^2^ test. If heterogeneity was considered relevant, e.g. I^2^ statistic greater than 0.50 and p<0.10, sources of heterogeneity were investigated, subanalyses were performed as deemed clinically relevant, and subtotals only, or single trial results were reported. In case of a meta-analysis, publication bias was assessed by drawing a funnel plot based on the primary outcome from all trials and statistical analysis of risk ratios or odds ratios as the measure of treatment effect.

A content analysis was conducted on the characteristics of the employed ATs, the target populations and the reported or hypothesized working mechanisms.

### Quality of evicence

Quality (or certainty) of evidence of the studies with significant outcomes only was was assessed with the Grading of Recommendations Assessment, Development and Evaluation (GRADE) [49]. Evidence can be scored as high, moderate, low or very low, according to a set of criteria.

## Results

### Study selection

The search yielded 776 unique citations. Based on title and abstract, 760 citations were excluded because the language was not English, Dutch or German (n = 23), were not about anxiety (n = 164), or it concerned anxiety related to another primary disease or condition (n = 175), didn’t concern adults (18–65 years) (n = 152), were not about AT (n = 94), were not a controlled trial (n = 131), or were lacking a control group (n = 22) or anxiety symptoms were not used as outcome measure (n = 1).

**Fig 1:**
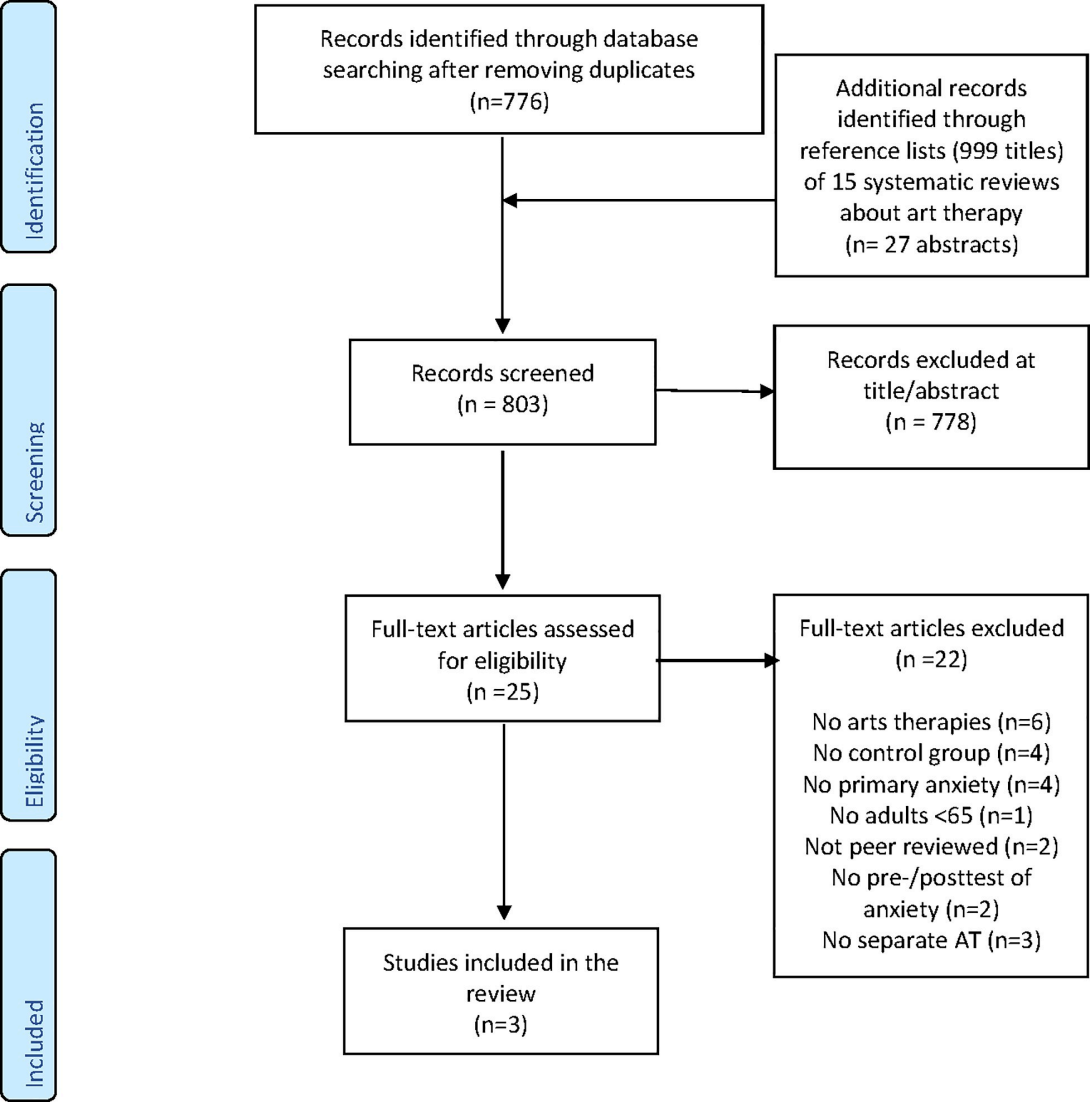
PRISMA flow diagram.

Of the remaining 16 full text articles, 13 articles were excluded. Reasons were: lack of a control group [50–54], anxiety was related to another primary disease or condition [55, 56], or the study population consisted of healthy subjects [57, 58], did not concern subjects in the age between 18–65 years [59], or was not peer-reviewed [60] or did not have pre-post measures of anxiety symptom severity [61, 62]. A list of all potentially relevant studies that were excluded from the review after reading full-texts, is presented in S2 Table *Excluded studies with reasons for exclusion*. Finally, three studies were included for the systematic review (Fig 1).

#### Screening of references from systematic reviews

The systematic literature search yielded 15 systematic reviews. All titles from the reference lists of these reviews were screened (n = 999), of which 27 publications were eligible for abstract screening and were other than the 938 citations found in the search described above (see Study selection). From these abstracts, 18 were excluded because they were not peer reviewed (n = 3), not in English, Dutch or German (n = 1), not about anxiety (n = 2), or were about anxiety related to cancer (n = 2), were not about AT (n = 2) or were not a controlled trial (n = 8). Nine full texts were screened for eligibility and were all excluded. Six full texts were excluded because these concerned psychodynamic therapies and did not include AT [63–68]. Two full texts were excluded because they concerned multidisciplinary treatment and no separate effects of AT were measured [18, 19]. The final full text was excluded because it concerned induced worry in a healthy population [69]. No studies remained for quality appraisal and full review. The justified reasons for exclusion of all potentially relevant studies that were read in full-text form, is presented in S2 Table *Excluded studies with reasons for exclusion*.

### Study characteristics

The review includes three RCTs. The study populations of the included studies are: students with PTSD symptoms and two groups of adults with fear for a specific situation: students prior to exams and prisoners prior to release. The trials have small to moderate sample sizes, ranging from 36 to 69. The total number of patients in the included studies is 162 (Table 1).

**Table 1:** Characteristics of the included studies of art therapy.

Study author & year	Funding source	Aim	Study type	Number/ (treated/control)	Study population
**Henderson et al. (2007)[70]**	Not provided	To examine the healing aspects of creating mandalas on mental health (anxiety and PTSD symptoms).	RCT	36 (19/17)	Sex: male and female Age:18–23 Population: **undergraduate students** (US) with PTSD symptoms Exclusion: simultaneous psychotherapy or phychotropic medication
**Sandmire et al. (2012)[71]**	Not provided	To assess if art making leads to (significant) anxiety reduction, compared to a control group.	RCT	57 (29/28)	Sex: male and female Age: 18.8 (mean) Population: **undergraduate first year students** of a liberal arts college (US), a week prior to final exams Exclusion: anxiety disorder, use of medication known to influence the central nervous system (e.g. for depression or ADHD)
**Yu et al. (2016) [72]**	Not provided	To examine the feasibility of using HTP drawing therapy as an intervention to reduce prisoners' prelease anxiety.	RCT	69 (33/36)	Sex: male Age: 18–60 Population: **prisoners** (China), to be released within the next 2 to 3 Months Diagnosis: none

In one study, AT is combined with another treatment: a group interview [72]. The other two studies solely concern AT (Table 2) [70, 71].

**Table 2:** Characteristics of the interventions of included studies.

Study author & year	Art therapy characteristics	Treatment duration, frequency, type (group or individual)	Co-interventions	Control group characteristics
**Henderson et al. (2007)**	Mandala creation (represent feelings or emotions related to personal trauma within the contour of a circle).	3 sessions; 20 minutes per session; 3 consecutive days (1 week); group therapy.	None	Active control: Three specific drawing assignments (not trauma related) of 20 minutes each.
**Sandmire et al. (2012)**	One choice out of five art- making activities: mandala design, free painting, collage making, free clay work and still life drawing; social interaction was allowed, no use of electronic devices.	1 session; 30 minutes; group therapy.	None	Inactive control: Sitting in comfortable chairs, social interaction was allowed, no use of electronic devices
**Yu et al. (2016)**	Drawing of at least a house, a tree and a person, followed by a group interview.	10 sessions; no set time for drawing; twice a week (5 weeks); group therapy; followed by group interview.	Group interview 40–60 min, 10 sessions, twice a week for 5 weeks.	Active control: Only group interview (40–60 min) twice a week over a period of 5 weeks

The provided AT varies considerably: mandala creation in which the trauma is represented [70] or colouring a pre-designed mandala, free clay work, free form painting, collage making, still life drawing [71], and house-tree-person drawings (HTP) [72]. Session duration differs from 20 minutes to 75 minutes. The therapy period ranges from only once to eight weeks, with one to ten sessions in total (Table 2). In one study, the control group receives the co-intervention only: group interview in Yu et al. [72]. Henderson et al. [70] use three specific drawing assignments as control condition, which are not focussed on trauma, opposed to the provided art therapy in the experimental group. Sandmire et al. [71] used inactive treatment. Here, AT is compared to comfortably sitting. Study settings were outpatient: universities (US) and prison (China). None of the RCTs reported on sources of funding for the studies.

See S3 Table for an extensive overview of characteristics and outcomes of the included studies.

### Risk of bias within studies

Based on the Cochrane Collaboration’s tool for assessing risk of bias, estimations of bias were made. Table 3 shows that the risk of bias (RoB) is high in all studies.

**Table 3:** Summary of risk of bias (high, low, unclear).

Study	Selection bias (risk on incomparable groups, due to sequence generation and allocation concealment)	Performance bias (blinding participants and therapists)	Detection bias (blinding outcome (assessors))	Attrition bias (incomplete outcome data)	Reporting bias (selective reporting)	Overall risk of bias
**Henderson et al. (2007)**	Unclear	High	Unclear	Unclear	Low	**High**
**Sandmire et al. (2012)**	Unclear	High	Unclear	Low	Low	**High**
**Yu et al. (2016)**	Unclear	High	Low	Low	Low	**High**

*Selection bias*: overall, methods of randomization were not always described and selection bias can therefore not be ruled out, which leads to unclear RoB. Henderson et al. [70] described the randomisation of participants over experimental and control groups. However, it is unclear how gender and type of trauma are distributed. Sandmire et al. [71] did not describe the randomization method but there was no baseline imbalance. Also Yu et al. [72] did not decribe the randomisation method, but two comparable groups were formed as concluded on baseline measures. Nevertheless it is unclear whether psychopathology of control and experimental groups are comparable.

*Performance bias*: Sandmire’s RCT had inactive control, which gives a high risk on performance bias [48]. Like in psychotherapy outcome research, blinding of patients and therapists is not feasible in AT [48, 73]. It is not possible to judge whether the lack of blinding influenced the outcomes and also none of the studies assessed treatment expectancies or credibility prior to or early in treatment, so all studies were scored as ‘high risk’ on performance bias.

*Detection bias*: in all studies only self-report questionnaires were used. The questionnaires used are all validated, which allows a low risk score of response bias. However, the exact circumstances under which measures are used are not described [70, 71] and may have given rise to bias. Presence of the therapist and or fear for lack of anonymity may have influenced scores and may have led to confirmation bias (e.g.[74]), which results in a ‘unclear’ risk of detection bias.

*Attrition bias*: in the study of Henderson it is not clear whether the outcome dataset is complete.

*Reporting bias*: there are no reasons to expect that there has been selective reporting in the studies.

*Other issues*: in Sandmire et al. [71] it was noted that the study population constists of liberal arts students, who are likely to have positive feelings towards art making and might expericence more positive effects (reduction of anxiety) than students from other disciplines.

*Overall risk of bias*: since all studies had one or more domains with high RoB, the overall RoB was high.

### Outcomes of individual studies

The measures used in the studies are shown in Table 4. The outcome measures for anxiety differ and include the State-Trait Anxiety Inventory (STAI) (used in two studies), the Hamilton Anxiety Rating Scale (HAM-A) and the Zung Self-rating Anxiety Scale (SAS) (used in one study). Quality of life was not measured in any of the included studies.

**Table 4:** Outcomes and summary of findings from the included studies.

Study author & year	Outcome measures	Time points	Intervention(s) and comparator	Significance of outcomes *between-groups*	Significance of outcomes *within-groups*
**Henderson et al. (2007)**	Anxiety: STAI	Pre- and post-treatment and follow-up (1 month later)	Experimental group (trauma-related mandala design; n = 19) *vs* control group (object drawing; n = 17)	Anxiety: NS	Exp. group: Anxiety: NS Control group: Anxiety (STAI): NS
**Sandmire et al. (2012)**	Anxiety: STAI	Pre- and post-art-making (no follow-up)	Experimental group (art-making; n = 29) vs control group (sitting; n = 28)	Anxiety (state): S** Anxiety (trait):S*	Exp. group: Anxiety (state): S** Anxiety (trait) S** Control group (inactive): Anxiety (state): NS Anxiety (trait): NS
**Zhan Yu et al. (2016)**	Anxiety: HAM-A SAS	Pre- and post-treatment (no follow-up)	Experimental group (HTP followed by group interview; n = 33) *vs* control group (only group interview; n = 36)	NR	Exp. group: HAM-A:S*** SAS:S** Control group: HAM-A: NS SAS:S* (higher anxiety score)

NR = Not reported.

NS = Not significant.

S = Significant

^*^ = p<0.05.

^**^ = p<0.01.

^***^ = p<0.001.

STAI: Spielberger's State-Trait Anxiety Inventory (self-report); HAM-A: Hamilton Anxiety Scale; SAS: Zung Self-Rating Anxiety Scale.

#### Anxiety–in study with inactive control

Sandmire et al. [71] showed significant between-group effects of art making on state anxiety (tested with ANOVA: experimental group (mean (SD)): 39.3 (9.4) - 29.5 (8.6); control group (mean (SD)): 36.2 (8.8) - 36.0 (10.9)\; *p* = 0.001) and on trait anxiety (experimental group (mean (SD)): 39.1 (5.8) - 33.3 (6.1); control group (mean (SD)): 38.2 (10.2) - 37.3 (11.2); *p* = 0.004) There were no significant differences in effectiveness between the five types of art making activities.

#### Anxiety–in studies with active control

Henderson et al. [70] reported no significant effect of creating mandalas (trauma-related art making) versus random art making on anxiety symptoms (tested with ANCOVA: experimental group (mean (SD)): 45.05 (10.75) - 41.16 (11.30); control group (mean (SD): 49.05 (12.29) - 44.05 (10.12), *p*-value: not reported) immediately after treatment. At follow-up after one month there was also no significant effect of creating mandalas on anxiety symptoms: experimental group (mean (SD): 40.95 (11.54); control group (mean (SD): 42.0 (13.26)), but there was significant improvement of PTSD symptom severity at one-month follow-up (*p* = 0.015).

Yu et al. (2016) did not report analyses of between-group effects. Only the experimental group, who made HTP drawings followed by group interview, showed a significant pre- versus post-treatment reduction of anxiety symptoms (two-tailed paired sample t-tests: HAM-A (mean (SD): 24.36 (9.11) - 17.42 (10.42), p = 0.001; SAS (mean (SD): 62.63 (9.46) - 56.78 (11.64,) *p* = 0.004). The anxiety level in the control group on the other hand, who received only group interview, increased between pre- and post-treatment (HAM-A (mean (SD): 24.75 (6.14) - 25.22 (7.37), not significant; SAS (mean (SD): 62.57 (7.36) - 66.11 (10.41), *p* = 0.33).

#### Summary of outcomes and quality

Of three included RCTs studying the effects of AT on reducing anxiety symptoms, one RCT [71] showed a significant anxiety reduction, one RCT [72] was inconclusive because no between-group outcomes were provided, and one RCT [70] found no significant anxiety reduction, but did find signifcant reduction of PTSD symptoms at follow-up.

Regarding within-group differences, two studies [71, 72] showed significant pre-posttreatment reduction of anxiety levels in the AT groups and one did not [70].

The quality of the evidence in Sandmire [71] as assessed with the GRADE classification is low to very low (due to limited information the exact classification could not be determined). The crucial risk of bias, which is likely to serious alter the results [49], combined the with small sample size (imprecision [75]) led to downgrading of at least two levels.

#### Meta-analysis

Because data were insufficiently comparable between the included studies due to variation in study populations, control treatments, the type of AT employed and the use of different measures, a meta-analysis was not performed.

### Narrative synthesis

#### Benefiting populations

AT seems to be effective in the treatment of pre-exam anxiety (for final exams) in adult liberal art students [71], although the quality of evidence is low due to high RoB. Based on pre-posttreatment anxiety reduction (within-group analysis) AT may be effective for adult prisoners with pre-release anxiety [72].

#### Characteristics of AT for anxiety

Sandmire et al. [71] gave students with pre-exam stress one choice out of five art-making activities: mandala design, free painting, collage making, free clay work or still life drawing. The activity was limited to one session of 30 minutes. This was done in a setting simulating an art center where students could use art materials to relieve stress. The mandala design activity consisted of a pre-designed mandala which could be completed by using pencils, tempera paints, watercolors, crayons or markers. The free form painting activity was carried out on a sheet of white paper using tempera or water color paints which were used to create an image from imagination. Participants could also use fine-tip permanent makers, crayons, colored pencils and pastels to add detailed design work upon completion of the initial painting. Collage making was also one of the five options. This was done with precut images and text, by further cutting out the images and additonal images from provided magazins and gluing them on a white piece of paper. Participants could also choose for a clay activity to make a ‘pleasing form’. Examples were a pinch pot, coil pot and small animal figures. The final option for art-making was a still life drawing, by arranging objects into a pleasing assembly and drafting with pencil. Additionally, diluted sepia ink could be used to paint in tonal values.

Yu et al. [72] used the HTP drawings in combination with group interviews about the drawings, to treat pre-release anxiety in male prisoners. The procedure consists of drawing a house, a tree and a person as well as some other objects on a sheet of paper. Yu follows the following interpretation: the house is regarded as the projection of family, the tree represents the environment and the person represents self-identification [76]. The HTP drawing is usually used as a diagnostic tool, but is used in this study as an intervention to enable prisoners to become more aware of their emotional issues and cognitions in relation to their upcoming release. A counselor gives helpful guidance based on the drawing and reflects on informal or missing content, so that the drawings can be enriched and completed. After completion of the drawings, prisoners participated in a group interview in which the unique attributes of the drawings are related to their personal situation and upcoming release.

Henderson et al. [70] treated traumatised students with mandala creation, aiming for the expression and representation of feelings. The participants were asked to draw a large circle and to fill the circle with feelings or emotions related to their personal trauma. They could use symbols, patterns, designs and colors, but no words. One session lasted 20 minutes and the total intervention consisted of three sessions, on three consecutive days. One month after the intervention, the participants were asked about the symbolic meaning of the mandala drawings.

#### Working mechanisms of AT

Sandmire used a single administration of art making to treat the handling of stressful situations (final exams) of undergraduate liberal art students. The art intervention did not explicitly expose students to the source of stress, hence a general working mechanism of AT is expected. The authors claim that art making offers a bottom-up approach to reduce anxiety. Art making, in a non-verbal, tactile and visual manner, helps entering a flow-like-state of mind that can reduce anxiety [77], comparable to mindfulness.

Yu reports that nonverbal symbolic methods, like HTP-drawing, are thought to reflect subconscious self-relevant information. The process of art making and reflection upon the art may lead to insights in emotions and (wrong) cognitions that can be addressed during counseling. The authors state that “HTP-drawing is a natural, easy mental intervention technique through which counselors can guide prisoners to form helpful cognitions and behaviors within a relative relaxing and well-protected psychological environment”. In this case the artwork is seen as a form of unconscious self-expression that opens up possibilities for verbal reflections and counseling. In the process of drawing, the counselor gives guidance so the drawing becomes more complete and enriched, what possibly entails a positive change in the prisoners’ cognitive patters and behavior.

Henderson treated PTSD symptoms in students and expected the therapy to work on anxiety symptoms as well. The AT intervention focussed on the creative expression of traumatic memories, which can been seen as an indirect approach to exposure, with active engagement. The authors indicate that mandala creation (related to trauma) leads to changes in cognition, facilitating increasing gains. Exposure, recall and emotional distancing may be important attributes to recovery.

Summarizing, three different types of AT can be distinguised: 1) using art-making as a pleasant and relaxing activity; 2) using art-making for expression of (unconsious) cognitive patterns, as an insightful tool; and 3) using the art-making process as a consious expression of difficult emotions and (traumatic) memories.

Based on these findings, we can hypothesize that AT may contribute to reducing anxiety symptom severity, because AT may:

- induce relaxation, by stimulating a flow-like state of mind, presumably leading to a reduction of cortisol levels and hence stress and anxiety reduction (stress regulation) [71];
- make the unconscious visible and thereby creating possibilities to investigate emotions and cognitions, contributing to cognitive regulation [70, 72].
- create a safe environment for the conscious expression of (difficult) emotions and memories, what is similar to exposure, recall and emotional distancing, possibly leading to better emotion regulation [70].

## Discussion

Currently there is no overview of evidence of effectiveness of AT on the reduction of anxiety symptoms and no overview of the intervention characteristics, the populations that might benefit from this treatment and the described and/ or hypothesized working mechanisms. Therefore, a systematic review was performed on RCTs and nRCTs, focusing on the effectiveness of AT in the treatment of anxiety in adults.

### Summary of evidence and limitations at study level

Three publications out of 776 hits of the search met all inclusion and exclusion criteria. No supplemented publications from the reference lists (999 titles) of 15 systematic reviews on AT could be included. Considering the small amount of studies, we can conclude that effectiveness research on AT for anxiety in adults is in a beginning state and is developing.

The included studies have a high risk of bias, small to moderate sample sizes and in total a very small number of patients (n = 162). As a result, there is no moderate or high quality evidence of the effectiveness of AT on reducing anxiety symptom severity. Low to very low-quality of evidence is shown for AT for pre-exam anxiety in undergraduate students [71]. One RCT on prelease anxiety in prisoners [72] was inconclusive because no between-group outcome analyses were provided, and one RCT on PTSD and anxiety symptoms in students [70] found significant reduction of PTSD symtoms at follow-up, but no significant anxiety reduction. Regarding within-group differences, two studies [71, 72] showed significant pre-posttreatment reduction of anxiety levels in the AT groups and one did not [70]. Intervention characteristics, populations that might benefit from this treatment and working mechanisms were described. In conclusion, these findings lead us to expect that art therapy may be effective in the treatment of anxiety in adults as it may improve stress regulation, cognitive regulation and emotion regulation.

### Strengths and limitations of this review

The strength of this review is firstly that it is the first systematic review on AT for primary anxiety symptoms. Secondly, its quality, because the Cochrane systematic review methodology was followed, the study protocol was registered before start of the review at PROSPERO, the AMSTAR 2 checklist was used to assess and improve the quality of the review and the results were reported according to the PRISMA guidelines. A third strength is that the search strategy covers a long period of 20 years and a large number of databases (13) and two journals.

A first limitation, according to assessment with the AMSTAR 2 checklist, is that only peer reviewed publications were included, which entails that many but not all data sources were included in the searches. Not included were searches in trial/study registries and in grey literature, since peer reviewed publication was an inclusion criterion. Content experts in the field were also not consulted. Secondly, only three RCTs met the inclusion criteria, each with a different target population: students with moderate PTSD, students with pre-exam anxiety and prisoners with pre-release anxiety. This means that only a small part of the populations of adults with anxiety (disorders) could be studied in this review. A third (possible) limitation concerns the restrictions regarding the included languages and search period applied (1997- October 2017). With respect to the latter it can be said that all included studies are published after 2006, making it likely that the restriction in search period has not influenced the outcome of this review. No studies from 1997 to 2007 met the inclusion and exclusion criteria. This might indicate that (n)RCTs in the field of AT, aimed at anxiety, are relatively new. A fourth limitation is the definition of AT that was used. There are many definitions for AT and discussions about the nature of AT (e.g. [78]). We considered an intervention to be *art therapy* in case the visual arts were used to promote health/wellbeing and/or the author called it art therapy. Thus, only art making as an artistic activity was excluded. This may have led to unwanted exclusion of interesting papers.

A fifth limitation is the use of the GRADE approach to assess the quality of evidence of art therapy studies. This tool is developed for judging quality of evidence of studies on pharmacological treatments, in which blinding is feasible and larger sample sizes are accustomed. However the assessed study was a RCT on art therapy [71], in which blinding of patients and therapists was not possible. Because the GRADE approach is not fully tailored for these type of studies, it was difficult to decide whether the the exact classification of the available evidence was low or very low.

### Comparison to the AT literature

The results of the review are in agreement with other findings in the scientific literature on AT demonstrating on the one hand promising results of AT and on the other hand showing many methodological weaknesses of AT trials. For example, other systematic reviews on AT also report on promising results for art therapy for PTSD [31–34, 37] and for a broader range of (mental) health conditions [35–39], but since these reviews also included lower quality study designs next to RCTs and nRCTs, the quality of this evidence is likely to be low to very low as well. These reviews also conclude on methodological shortcomings of art therapy effectiveness studies.

Three approaches in AT were identified in this review: 1) using art-making as a relaxing activity, leading to stress reduction; 2) using the art-making process as a consious pathway to difficult emotions and (traumatic) memories; leading to better emotion regulation; and 3) using art-making for expression, to gain insight in (unconscious) cognitive patterns; leading to better cognitive regulation.

These three approaches can be linked to two major directions in art therapy, identified by Holmqvist & Persson [74]: “art-as-therapy” and “art-in-psychotherapy”. *Art-as-therapy* focuses on the healing ability and relaxing qualities of the art process itself and was first described by Kramer in 1971 [79]. This can be linked to the findings in the study of Sandmire [71], where it is suggested that art making led to lower stress levels. Art making is already associated with lower cortisol levels [80]. A possible explanation for this finding can be that a trance-like state (in flow) occurs during art-making [81] due to the tactile and visual experience as well as the repetitive muscular activity inherent to art making.

*Art-in-psychotherapy*, first described by Naumberg [82] encompasses both the unconscious and the conscious (or semi-conscious) expression of inner feelings and experiences in apparently free and explicit exercises respectively. The art work helps a patient to open up towards their therapist [74], so what the patient experienced during the process of creating the art work, can be deepened in conversation. In practice, these approaches often overlap and interweave with one another [83], which is probably why it is combined in one direction ‘art-in-psychotherapy’. It might be beneficial to consider these ways of conscious and unconscious expression separately, because it is a fundamental different view on the importance of art making.

The overall picture of the described and hypothesized working mechanisms that emerged in this review lead to the hypotheses that anxiety symptoms may decrease because AT may support stress regulation (by inducing relaxation, presumably comparable to mindfulness [64,84], emotion regulation (by creating the safe condition for expression and examination of emotions) and cognitive regulation (as art work opens up possibilities to investigate (unconscious) cognitions). These types of regulation all contribute to better self-regulation [85]. The hypothesis with respect to stress regulation is further supported by results from other studies. The process of creating art can promote a state of mindfulness [57]. Mindfulness can increase self-regulation [84] which is a moderator between coping strength and mental symptomatology [86]. Improving patient’s self-regulation leads, amongst others, to improvement of coping with disease conditions like anxiety [85, 86]. Our findings are in accordance with the findings of Haeyen [30], stating that patients learn to express emotions more effectively, because AT enables them to “examine feelings without words, pre-verbally and sometimes less consciously”, (p.2). The connection between art therapy and emotion regulation is also supported by the recently published narrative review of Gruber & Oepen [87], who found significant effective short-term mood repair through art making, based on two emotion regulation strategies: venting of negative feelings and distraction strategy: attentional deployment that focuses on positive or neutral emotions to distract from negative emotions.

### Future perspectives

Even though this review cannot conclude effectiveness of AT for anxiety in adults, that does not mean that AT does not work. Art therapists and other care professionals do experience the high potential of AT in clinical practice. It is challenging to find ways to objectify these practical experiences.

The results of the systematic review demonstrate that high quality trials studying effectiveness and working mechanisms of AT for anxiety disorders in general and specifically, and for people with anxiety in specific situations are still lacking. To get high quality evidence of effectiveness of AT on anxiety (disorders), more robust studies are needed.

Besides anxiety symptoms, the effectiveness of AT on aspects of self-regulation like emotion regulation, cognitive regulation and stress regulation should be further studied as well. By evaluating the changes that may occur in the different areas of self-regulation, better hypotheses can be generated with respect to the working mechanisms of AT in the treatment of anxiety.

A key point for AT researchers in developing, executing and reporting on RCTs, is the issue of risk of bias. It is recommended to address more specifically how RoB was minimalized in the design and execution of the study. This can lower the RoB and therefor enhance the quality of the evidence, as judged by reviewers. One of the scientific challenges here is how to assess performance bias in AT reviews. Since blinding of therapists and patients in AT is impossible, and if performance bias is only considered by ‘lack of blinding of patients and personnel’, every trial on art therapy will have a high risk on performance bias, making the overall RoB high. This implies that high or even medium quality of evidence can never be reached for this intervention, even when all other aspects of the study are of high quality. Behavioral interventions, like psychotherapy and other complex interventions, face the same challenge. In 2017, Munder & Barth [48] published considerations on how to use the Cochrane's risk of bias tool in psychotherapy outcome research. We fully support the recommendations of Grant and colleagues [73] and would like to emphasize that tools for assessing risk of bias and quality of evidence need to be tailored to art therapy and (other) complex interventions where blinding is not possible.

## Conclusions

The effectiveness of AT on reducing anxiety symptoms severity has hardly been studied in RCTs and nRCTs. There is low-quality to very low-quality evidence of effectiveness of AT for pre-exam anxiety in undergraduate students. AT may also be effective in reducing pre-release anxiety in prisoners.

The included RCTs demonstrate a wide variety in AT characteristics (AT types, numbers and duration of sessions). The described or hypothesized working mechanisms of art making are: induction of relaxation; working on emotion regulation by creating the safe condition for conscious expression and exploration of difficult emotions, memories and trauma; and working on cognitive regulation by using the art process to open up possibilities to investigate and (positively) change (unconscious) cognitions, beliefs and thoughts.

High quality trials studying effectiveness on anxiety and mediating working mechanisms of AT are currently lacking for all anxiety disorders and for people with anxiety in specific situations.

## Supporting information

S1 Checklist PRISMA checklist. (PDF)

S1 File Full list of search terms and databases. (PDF)

S1 Table Data extraction form. (PDF)

S2 Table Excluded studies with reasons for exclusion. (PDF)

S3 Table Background characteristics of the included studies. (PDF)
