# Supplementary material for: The effectiveness of art therapy for anxiety in adults: A systematic review of randomised and non-randomised controlled trials
Source: PLoS One. 2018 Dec 17;13(12):e0208716. doi: 10.1371/journal.pone.0208716 (PMC6296656; doi:10.1371/journal.pone.0208716)
Supplement: S1 File — (PDF) [file pone.0208716.s002.pdf]

APPENDIX I - Search Strategy

PubMed

(("Sensory Art Therapies"[Mesh:noexp] OR "Art Therapy"[mesh] OR "Color Therapy"[mesh] OR "art therapy"[tw] OR "arts therapy"[tw] OR "art therapies"[tw] OR "arts therapies"[tw] OR "visual art therapy"[tw] OR "visual arts therapy"[tw] OR "Color Therapy"[tw] OR "Color Therapies"[tw] OR "Colour Therapy"[tw] OR "Colour Therapies"[tw] OR "painting therapy"[tw] OR "drawing therapy"[tw] OR "sculpting therapy"[tw] OR "clay therapy"[tw] OR "clay sculpting"[tw] OR "sculpturing therapy"[tw] OR "clay sculpturing"[tw] OR clay model*[tw]) AND ("Anxiety Disorders"[Mesh] OR "anxiety disorder"[tw] OR "anxiety disorders"[tw] OR "Anxiety Neuroses"[tw] OR "Anxiety Neurosis"[tw] OR "Neurotic Anxiety State"[tw] OR "Neurotic Anxiety States"[tw] OR "Agoraphobia"[tw] OR "Separation Anxiety"[tw] OR "Neurocirculatory Asthenia"[tw] OR "Neurotic Disorders"[tw] OR "Obsessive-Compulsive Disorder"[tw] OR "Hoarding Disorder"[tw] OR "Panic Disorder"[tw] OR "Phobic Disorders"[tw] OR "Social Phobia"[tw] OR "Neurotic Disorder"[tw] OR "Obsessive-Compulsive Disorders"[tw] OR "OCD"[tw] OR "Hoarding Disorders"[tw] OR "Panic Disorders"[tw] OR "Phobic Disorder"[tw] OR "Social Phobias"[tw] OR "phobia"[tw] OR "phobias"[tw] OR "Stress Disorders, Post-Traumatic"[Mesh] OR "PTSD"[tw] OR "post-traumatic stress disorder"[tw] OR "post-traumatic stress disorders"[tw] OR "Posttraumatic Stress Disorders"[tw] OR "Posttraumatic Stress Disorder"[tw] OR "Posttraumatic Neuroses"[tw] OR "Posttraumatic Neurosis"[tw] OR "Post-Traumatic Neuroses"[tw] OR "Post-Traumatic Neurosis"[tw] OR "Stress Disorders, Traumatic"[Mesh] OR "Traumatic Stress Disorder"[tw] OR "Traumatic Stress Disorders"[tw] OR "Battered Child Syndrome"[tw] OR "Combat Disorders"[tw] OR "Psychological Trauma"[tw] OR "Combat Disorder"[tw] OR "Psychological Traumas"[tw] OR psychotrauma*[tw] OR "generalized anxiety disorder"[tw] OR "generalized anxiety disorders"[tw] OR "GAD"[tw] OR "panic"[tw] OR "Anxiety"[mesh] OR anxiet*[tw]))

Embase

(("Art Therapy"/ OR "Color Therapy"/ OR "art therapy".mp OR "arts therapy".mp OR "art therapies".mp OR "arts therapies".mp OR "visual art therapy".mp OR "visual arts therapy".mp OR "Color Therapy".mp OR "Color Therapies".mp OR "Colour Therapy".mp OR "Colour Therapies".mp OR "painting therapy".mp OR "drawing therapy".mp OR "sculpting therapy".mp OR "clay therapy".mp OR "clay sculpting".mp OR clay model*.mp OR "sculpturing therapy".mp OR "clay sculpturing".mp) AND (exp "Anxiety Disorder"/ OR "anxiety disorder".mp OR "anxiety disorders".mp OR "Anxiety Neuroses".mp OR "Anxiety Neurosis".mp OR "Neurotic Anxiety State".mp OR "Neurotic Anxiety States".mp OR "Agoraphobia".mp OR "Separation Anxiety".mp OR "Neurocirculatory Asthenia".mp OR "Neurotic Disorders".mp OR "Obsessive-Compulsive Disorder".mp OR "Hoarding Disorder".mp OR "Panic Disorder".mp OR "Phobic Disorders".mp OR "Social Phobia".mp OR "Neurotic Disorder".mp OR "Obsessive-Compulsive Disorders".mp OR "OCD".mp OR "Hoarding Disorders".mp OR "Panic Disorders".mp OR "Phobic Disorder".mp OR "Social Phobias".mp OR "phobia".mp OR "phobias".mp OR "posttraumatic stress disorder"/ OR "PTSD".mp OR "post-traumatic stress disorder".mp OR "post-traumatic stress disorders".mp OR "Posttraumatic Stress Disorders".mp OR "Posttraumatic Stress Disorder".mp OR "Posttraumatic Neuroses".mp OR "Posttraumatic Neurosis".mp OR "Post-Traumatic Neuroses".mp OR "Post-Traumatic Neurosis".mp OR "Traumatic Stress Disorder".mp OR "Traumatic Stress Disorders".mp OR "Battered Child Syndrome".mp OR "Combat Disorders".mp OR "Psychological Trauma".mp OR "Combat Disorder".mp OR

"psychotrauma"/ OR psychotrauma*.mp OR "Psychological Traumas".mp OR "generalized anxiety disorder "/ OR "generalized anxiety disorder".mp OR "generalized anxiety disorders".mp OR "GAD".mp OR "panic".mp OR exp "Anxiety"/ OR anxiet*.mp)) NOT (conference review or conference abstract).pt

Web of Science

TS=(("Art Therapy" OR "Color Therapy" OR "art therapy" OR "arts therapy" OR "art therapies" OR "arts therapies" OR "visual art therapy" OR "visual arts therapy" OR "Color Therapy" OR "Color Therapies" OR "Colour Therapy" OR "Colour Therapies" OR "painting therapy" OR "drawing therapy" OR "sculpting therapy" OR "clay therapy" OR "clay sculpting" OR clay model*) AND ("Anxiety Disorder" OR "anxiety disorder" OR "anxiety disorders" OR "Anxiety Neuroses" OR "Anxiety Neurosis" OR "Neurotic Anxiety State" OR "Neurotic Anxiety States" OR "Agoraphobia" OR "Separation Anxiety" OR "Neurocirculatory Asthenia" OR "Neurotic Disorders" OR "Obsessive-Compulsive Disorder" OR "Hoarding Disorder" OR "Panic Disorder" OR "Phobic Disorders" OR "Social Phobia" OR "Neurotic Disorder" OR "Obsessive-Compulsive Disorders" OR "OCD" OR "Hoarding Disorders" OR "Panic Disorders" OR "Phobic Disorder" OR "Social Phobias" OR "phobia" OR "phobias" OR "posttraumatic stress disorder" OR "PTSD" OR "post-traumatic stress disorder" OR "post-traumatic stress disorders" OR "Posttraumatic Stress Disorders" OR "Posttraumatic Stress Disorder" OR "Posttraumatic Neuroses" OR "Posttraumatic Neurosis" OR "Post-Traumatic Neuroses" OR "Post-Traumatic Neurosis" OR "Traumatic Stress Disorder" OR "Traumatic Stress Disorders" OR "Battered Child Syndrome" OR "Combat Disorders" OR "Psychological Trauma" OR "Combat Disorder" OR "psychotrauma" OR psychotrauma* OR "Psychological Traumas" OR "generalized anxiety disorder " OR "generalized anxiety disorder" OR "generalized anxiety disorders" OR "GAD" OR "panic" OR "Anxiety" OR anxiet*))

Cochrane

(("Art Therapy" OR "Color Therapy" OR "art therapy" OR "arts therapy" OR "art therapies" OR "arts therapies" OR "visual art therapy" OR "visual arts therapy" OR "Color Therapy" OR "Color Therapies" OR "Colour Therapy" OR "Colour Therapies" OR "painting therapy" OR "drawing therapy" OR "sculpting therapy" OR "clay therapy" OR "clay sculpting" OR clay model*) AND ("Anxiety Disorder" OR "anxiety disorder" OR "anxiety disorders" OR "Anxiety Neuroses" OR "Anxiety Neurosis" OR "Neurotic Anxiety State" OR "Neurotic Anxiety States" OR "Agoraphobia" OR "Separation Anxiety" OR "Neurocirculatory Asthenia" OR "Neurotic Disorders" OR "Obsessive-Compulsive Disorder" OR "Hoarding Disorder" OR "Panic Disorder" OR "Phobic Disorders" OR "Social Phobia" OR "Neurotic Disorder" OR "Obsessive-Compulsive Disorders" OR "OCD" OR "Hoarding Disorders" OR "Panic Disorders" OR "Phobic Disorder" OR "Social Phobias" OR "phobia" OR "phobias" OR "posttraumatic stress disorder" OR "PTSD" OR "post-traumatic stress disorder" OR "post-traumatic stress disorders" OR "Posttraumatic Stress Disorders" OR "Posttraumatic Stress Disorder" OR "Posttraumatic Neuroses" OR "Posttraumatic Neurosis" OR "Post-Traumatic Neuroses" OR "Post-Traumatic Neurosis" OR "Traumatic Stress Disorder" OR "Traumatic Stress Disorders" OR "Battered Child Syndrome" OR "Combat Disorders" OR "Psychological Trauma" OR "Combat Disorder" OR "psychotrauma" OR psychotrauma* OR "Psychological Traumas" OR "generalized anxiety disorder " OR "generalized anxiety disorder" OR "generalized anxiety disorders" OR "GAD" OR "panic" OR "Anxiety" OR anxiet*))

CENTRAL

(("Art Therapy" OR "Color Therapy" OR "art therapy" OR "arts therapy" OR "art therapies" OR "arts therapies" OR "visual art therapy" OR "visual arts therapy" OR "Color Therapy" OR "Color Therapies" OR "Colour Therapy" OR "Colour Therapies" OR "painting therapy" OR "drawing therapy" OR "sculpting therapy" OR "clay therapy" OR "clay sculpting" OR clay model*) AND ("Anxiety Disorder" OR "anxiety disorder" OR "anxiety disorders" OR "Anxiety Neuroses" OR "Anxiety Neurosis" OR "Neurotic Anxiety State" OR "Neurotic Anxiety States" OR "Agoraphobia" OR "Separation Anxiety" OR "Neurocirculatory Asthenia" OR "Neurotic Disorders" OR "Obsessive-Compulsive Disorder" OR "Hoarding Disorder" OR "Panic Disorder" OR "Phobic Disorders" OR "Social Phobia" OR "Neurotic Disorder" OR "Obsessive-Compulsive Disorders" OR "OCD" OR "Hoarding Disorders" OR "Panic Disorders" OR "Phobic Disorder" OR "Social Phobias" OR "phobia" OR "phobias" OR "posttraumatic stress disorder" OR "PTSD" OR "post-traumatic stress disorder" OR "post-traumatic stress disorders" OR "Posttraumatic Stress Disorders" OR "Posttraumatic Stress Disorder" OR "Posttraumatic Neuroses" OR "Posttraumatic Neurosis" OR "Post-Traumatic Neuroses" OR "Post-Traumatic Neurosis" OR "Traumatic Stress Disorder" OR "Traumatic Stress Disorders" OR "Battered Child Syndrome" OR "Combat Disorders" OR "Psychological Trauma" OR "Combat Disorder" OR "psychotrauma" OR psychotrauma* OR "Psychological Traumas" OR "generalized anxiety disorder " OR "generalized anxiety disorder" OR "generalized anxiety disorders" OR "GAD" OR "panic" OR "Anxiety" OR anxiet*))

Emcare

(("Art Therapy"/ OR "Color Therapy"/ OR "art therapy".mp OR "arts therapy".mp OR "art therapies".mp OR "arts therapies".mp OR "visual art therapy".mp OR "visual arts therapy".mp OR "Color Therapy".mp OR "Color Therapies".mp OR "Colour Therapy".mp OR "Colour Therapies".mp OR "painting therapy".mp OR "drawing therapy".mp OR "sculpting therapy".mp OR "clay therapy".mp OR "clay sculpting".mp OR clay model*.mp OR "sculpturing therapy".mp OR "clay sculpturing".mp) AND (exp "Anxiety Disorder"/ OR "anxiety disorder".mp OR "anxiety disorders".mp OR "Anxiety Neuroses".mp OR "Anxiety Neurosis".mp OR "Neurotic Anxiety State".mp OR "Neurotic Anxiety States".mp OR "Agoraphobia".mp OR "Separation Anxiety".mp OR "Neurocirculatory Asthenia".mp OR "Neurotic Disorders".mp OR "Obsessive-Compulsive Disorder".mp OR "Hoarding Disorder".mp OR "Panic Disorder".mp OR "Phobic Disorders".mp OR "Social Phobia".mp OR "Neurotic Disorder".mp OR "Obsessive-Compulsive Disorders".mp OR "OCD".mp OR "Hoarding Disorders".mp OR "Panic Disorders".mp OR "Phobic Disorder".mp OR "Social Phobias".mp OR "phobia".mp OR "phobias".mp OR "posttraumatic stress disorder"/ OR "PTSD".mp OR "post-traumatic stress disorder".mp OR "post-traumatic stress disorders".mp OR "Posttraumatic Stress Disorders".mp OR "Posttraumatic Stress Disorder".mp OR "Posttraumatic Neuroses".mp OR "Posttraumatic Neurosis".mp OR "Post-Traumatic Neuroses".mp OR "Post-Traumatic Neurosis".mp OR "Traumatic Stress Disorder".mp OR "Traumatic Stress Disorders".mp OR "Battered Child Syndrome".mp OR "Combat Disorders".mp OR "Psychological Trauma".mp OR "Combat Disorder".mp OR "psychotrauma"/ OR psychotrauma*.mp OR "Psychological Traumas".mp OR "generalized anxiety disorder "/ OR "generalized anxiety disorder".mp OR "generalized anxiety disorders".mp OR "GAD".mp OR "panic".mp OR exp "Anxiety"/ OR anxiet*.mp))

PsycINFO

((DE "Creative Arts Therapy" OR DE "Art Therapy" OR DE "Painting (Art)" OR DE "Art" OR DE "Drawing" OR DE "Sculpturing") AND (DE "Anxiety Disorders" OR DE "Acute Stress Disorder" OR DE "Castration Anxiety" OR DE "Death Anxiety" OR DE "Generalized Anxiety Disorder" OR DE "Obsessive Compulsive Disorder" OR DE "Panic Disorder" OR DE "Phobias" OR DE "Post-Traumatic Stress" OR DE "Posttraumatic Stress Disorder" OR DE "Separation Anxiety Disorder" OR DE "Anxiety" OR DE "Computer Anxiety" OR DE "Mathematics Anxiety" OR DE "Performance Anxiety" OR DE "Social Anxiety" OR DE "Speech Anxiety" OR DE "Test Anxiety")) **OR** TI(("Art Therapy" OR "Color Therapy" OR "art therapy" OR "arts therapy" OR "art therapies" OR "arts therapies" OR "visual art therapy" OR "visual arts therapy" OR "Color Therapy" OR "Color Therapies" OR "Colour Therapy" OR "Colour Therapies" OR "painting therapy" OR "drawing therapy" OR "sculpting therapy" OR "clay therapy" OR "clay sculpting" OR clay model*) AND ("Anxiety Disorder" OR "anxiety disorder" OR "anxiety disorders" OR "Anxiety Neuroses" OR "Anxiety Neurosis" OR "Neurotic Anxiety State" OR "Neurotic Anxiety States" OR "Agoraphobia" OR "Separation Anxiety" OR "Neurocirculatory Asthenia" OR "Neurotic Disorders" OR "Obsessive-Compulsive Disorder" OR "Hoarding Disorder" OR "Panic Disorder" OR "Phobic Disorders" OR "Social Phobia" OR "Neurotic Disorder" OR "Obsessive-Compulsive Disorders" OR "OCD" OR "Hoarding Disorders" OR "Panic Disorders" OR "Phobic Disorder" OR "Social Phobias" OR "phobia" OR "phobias" OR "posttraumatic stress disorder" OR "PTSD" OR "post-traumatic stress disorder" OR "post-traumatic stress disorders" OR "Posttraumatic Stress Disorders" OR "Posttraumatic Stress Disorder" OR "Posttraumatic Neuroses" OR "Posttraumatic Neurosis" OR "Post-Traumatic Neuroses" OR "Post-Traumatic Neurosis" OR "Traumatic Stress Disorder" OR "Traumatic Stress Disorders" OR "Battered Child Syndrome" OR "Combat Disorders" OR "Psychological Trauma" OR "Combat Disorder" OR "psychotrauma" OR psychotrauma* OR "Psychological Traumas" OR "generalized anxiety disorder " OR "generalized anxiety disorder" OR "generalized anxiety disorders" OR "GAD" OR "panic" OR "Anxiety" OR anxiet*)) **OR** SU(("Art Therapy" OR "Color Therapy" OR "art therapy" OR "arts therapy" OR "art therapies" OR "arts therapies" OR "visual art therapy" OR "visual arts therapy" OR "Color Therapy" OR "Color Therapies" OR "Colour Therapy" OR "Colour Therapies" OR "painting therapy" OR "drawing therapy" OR "sculpting therapy" OR "clay therapy" OR "clay sculpting" OR clay model*) AND ("Anxiety Disorder" OR "anxiety disorder" OR "anxiety disorders" OR "Anxiety Neuroses" OR "Anxiety Neurosis" OR "Neurotic Anxiety State" OR "Neurotic Anxiety States" OR "Agoraphobia" OR "Separation Anxiety" OR "Neurocirculatory Asthenia" OR "Neurotic Disorders" OR "Obsessive-Compulsive Disorder" OR "Hoarding Disorder" OR "Panic Disorder" OR "Phobic Disorders" OR "Social Phobia" OR "Neurotic Disorder" OR "Obsessive-Compulsive Disorders" OR "OCD" OR "Hoarding Disorders" OR "Panic Disorders" OR "Phobic Disorder" OR "Social Phobias" OR "phobia" OR "phobias" OR "posttraumatic stress disorder" OR "PTSD" OR "post-traumatic stress disorder" OR "post-traumatic stress disorders" OR "Posttraumatic Stress Disorders" OR "Posttraumatic Stress Disorder" OR "Posttraumatic Neuroses" OR "Posttraumatic Neurosis" OR "Post-Traumatic Neuroses" OR "Post-Traumatic Neurosis" OR "Traumatic Stress Disorder" OR "Traumatic Stress Disorders" OR "Battered Child Syndrome" OR "Combat Disorders" OR "Psychological Trauma" OR "Combat Disorder" OR "psychotrauma" OR psychotrauma* OR "Psychological Traumas" OR "generalized anxiety disorder " OR "generalized anxiety disorder" OR "generalized anxiety disorders" OR "GAD" OR "panic" OR "Anxiety" OR anxiet*)) **OR** MA(("Art Therapy" OR "Color Therapy" OR "art therapy" OR "arts therapy" OR "art therapies" OR "arts therapies" OR "visual art therapy" OR "visual arts therapy" OR "Color

Therapy" OR "Color Therapies" OR "Colour Therapy" OR "Colour Therapies" OR "painting therapy" OR "drawing therapy" OR "sculpting therapy" OR "clay therapy" OR "clay sculpting" OR clay model*) AND ("Anxiety Disorder" OR "anxiety disorder" OR "anxiety disorders" OR "Anxiety Neuroses" OR "Anxiety Neurosis" OR "Neurotic Anxiety State" OR "Neurotic Anxiety States" OR "Agoraphobia" OR "Separation Anxiety" OR "Neurocirculatory Asthenia" OR "Neurotic Disorders" OR "Obsessive-Compulsive Disorder" OR "Hoarding Disorder" OR "Panic Disorder" OR "Phobic Disorders" OR "Social Phobia" OR "Neurotic Disorder" OR "Obsessive-Compulsive Disorders" OR "OCD" OR "Hoarding Disorders" OR "Panic Disorders" OR "Phobic Disorder" OR "Social Phobias" OR "phobia" OR "phobias" OR "posttraumatic stress disorder" OR "PTSD" OR "post-traumatic stress disorder" OR "post-traumatic stress disorders" OR "Posttraumatic Stress Disorders" OR "Posttraumatic Stress Disorder" OR "Posttraumatic Neuroses" OR "Posttraumatic Neurosis" OR "Post-Traumatic Neuroses" OR "Post-Traumatic Neurosis" OR "Traumatic Stress Disorder" OR "Traumatic Stress Disorders" OR "Battered Child Syndrome" OR "Combat Disorders" OR "Psychological Trauma" OR "Combat Disorder" OR "psychotrauma" OR psychotrauma* OR "Psychological Traumas" OR "generalized anxiety disorder " OR "generalized anxiety disorder" OR "generalized anxiety disorders" OR "GAD" OR "panic" OR "Anxiety" OR anxiet*)) **OR** AB(("Art Therapy" OR "Color Therapy" OR "art therapy" OR "arts therapy" OR "art therapies" OR "arts therapies" OR "visual art therapy" OR "visual arts therapy" OR "Color Therapy" OR "Color Therapies" OR "Colour Therapy" OR "Colour Therapies" OR "painting therapy" OR "drawing therapy" OR "sculpting therapy" OR "clay therapy" OR "clay sculpting" OR clay model*) AND ("Anxiety Disorder" OR "anxiety disorder" OR "anxiety disorders" OR "Anxiety Neuroses" OR "Anxiety Neurosis" OR "Neurotic Anxiety State" OR "Neurotic Anxiety States" OR "Agoraphobia" OR "Separation Anxiety" OR "Neurocirculatory Asthenia" OR "Neurotic Disorders" OR "Obsessive-Compulsive Disorder" OR "Hoarding Disorder" OR "Panic Disorder" OR "Phobic Disorders" OR "Social Phobia" OR "Neurotic Disorder" OR "Obsessive-Compulsive Disorders" OR "OCD" OR "Hoarding Disorders" OR "Panic Disorders" OR "Phobic Disorder" OR "Social Phobias" OR "phobia" OR "phobias" OR "posttraumatic stress disorder" OR "PTSD" OR "post-traumatic stress disorder" OR "post-traumatic stress disorders" OR "Posttraumatic Stress Disorders" OR "Posttraumatic Stress Disorder" OR "Posttraumatic Neuroses" OR "Posttraumatic Neurosis" OR "Post-Traumatic Neuroses" OR "Post-Traumatic Neurosis" OR "Traumatic Stress Disorder" OR "Traumatic Stress Disorders" OR "Battered Child Syndrome" OR "Combat Disorders" OR "Psychological Trauma" OR "Combat Disorder" OR "psychotrauma" OR psychotrauma* OR "Psychological Traumas" OR "generalized anxiety disorder " OR "generalized anxiety disorder" OR "generalized anxiety disorders" OR "GAD" OR "panic" OR "Anxiety" OR anxiet*))

ArtIndex

TX(("Art Therapy" OR "Color Therapy" OR "art therapy" OR "arts therapy" OR "art therapies" OR "arts therapies" OR "visual art therapy" OR "visual arts therapy" OR "Color Therapy" OR "Color Therapies" OR "Colour Therapy" OR "Colour Therapies" OR "painting therapy" OR "drawing therapy" OR "sculpting therapy" OR "clay therapy" OR "clay sculpting" OR clay model*) AND ("Anxiety Disorder" OR "anxiety disorder" OR "anxiety disorders" OR "Anxiety Neuroses" OR "Anxiety Neurosis" OR "Neurotic Anxiety State" OR "Neurotic Anxiety States" OR "Agoraphobia" OR "Separation Anxiety" OR "Neurocirculatory Asthenia" OR "Neurotic Disorders" OR "Obsessive-Compulsive Disorder" OR "Hoarding Disorder" OR "Panic Disorder" OR "Phobic Disorders" OR "Social Phobia" OR "Neurotic Disorder" OR "Obsessive-Compulsive Disorders" OR "OCD" OR "Hoarding

Disorders" OR "Panic Disorders" OR "Phobic Disorder" OR "Social Phobias" OR "phobia" OR "phobias" OR "posttraumatic stress disorder" OR "PTSD" OR "post-traumatic stress disorder" OR "post-traumatic stress disorders" OR "Posttraumatic Stress Disorders" OR "Posttraumatic Stress Disorder" OR "Posttraumatic Neuroses" OR "Posttraumatic Neurosis" OR "Post-Traumatic Neuroses" OR "Post-Traumatic Neurosis" OR "Traumatic Stress Disorder" OR "Traumatic Stress Disorders" OR "Battered Child Syndrome" OR "Combat Disorders" OR "Psychological Trauma" OR "Combat Disorder" OR "psychotrauma" OR psychotrauma* OR "Psychological Traumas" OR "generalized anxiety disorder " OR "generalized anxiety disorder" OR "generalized anxiety disorders" OR "GAD" OR "panic" OR "Anxiety" OR anxiet*))

Academic Search Premier [fulltextzoeken]

TI(("Art Therapy" OR "Color Therapy" OR "art therapy" OR "arts therapy" OR "art therapies" OR "arts therapies" OR "visual art therapy" OR "visual arts therapy" OR "Color Therapy" OR "Color Therapies" OR "Colour Therapy" OR "Colour Therapies" OR "painting therapy" OR "drawing therapy" OR "sculpting therapy" OR "clay therapy" OR "clay sculpting" OR clay model*) AND ("Anxiety Disorder" OR "anxiety disorder" OR "anxiety disorders" OR "Anxiety Neuroses" OR "Anxiety Neurosis" OR "Neurotic Anxiety State" OR "Neurotic Anxiety States" OR "Agoraphobia" OR "Separation Anxiety" OR "Neurocirculatory Asthenia" OR "Neurotic Disorders" OR "Obsessive-Compulsive Disorder" OR "Hoarding Disorder" OR "Panic Disorder" OR "Phobic Disorders" OR "Social Phobia" OR "Neurotic Disorder" OR "Obsessive-Compulsive Disorders" OR "OCD" OR "Hoarding Disorders" OR "Panic Disorders" OR "Phobic Disorder" OR "Social Phobias" OR "phobia" OR "phobias" OR "posttraumatic stress disorder" OR "PTSD" OR "post-traumatic stress disorder" OR "post-traumatic stress disorders" OR "Posttraumatic Stress Disorders" OR "Posttraumatic Stress Disorder" OR "Posttraumatic Neuroses" OR "Posttraumatic Neurosis" OR "Post-Traumatic Neuroses" OR "Post-Traumatic Neurosis" OR "Traumatic Stress Disorder" OR "Traumatic Stress Disorders" OR "Battered Child Syndrome" OR "Combat Disorders" OR "Psychological Trauma" OR "Combat Disorder" OR "psychotrauma" OR psychotrauma* OR "Psychological Traumas" OR "generalized anxiety disorder " OR "generalized anxiety disorder" OR "generalized anxiety disorders" OR "GAD" OR "panic" OR "Anxiety" OR anxiet*)) OR SU(("Art Therapy" OR "Color Therapy" OR "art therapy" OR "arts therapy" OR "art therapies" OR "arts therapies" OR "visual art therapy" OR "visual arts therapy" OR "Color Therapy" OR "Color Therapies" OR "Colour Therapy" OR "Colour Therapies" OR "painting therapy" OR "drawing therapy" OR "sculpting therapy" OR "clay therapy" OR "clay sculpting" OR clay model*) AND ("Anxiety Disorder" OR "anxiety disorder" OR "anxiety disorders" OR "Anxiety Neuroses" OR "Anxiety Neurosis" OR "Neurotic Anxiety State" OR "Neurotic Anxiety States" OR "Agoraphobia" OR "Separation Anxiety" OR "Neurocirculatory Asthenia" OR "Neurotic Disorders" OR "Obsessive-Compulsive Disorder" OR "Hoarding Disorder" OR "Panic Disorder" OR "Phobic Disorders" OR "Social Phobia" OR "Neurotic Disorder" OR "Obsessive-Compulsive Disorders" OR "OCD" OR "Hoarding Disorders" OR "Panic Disorders" OR "Phobic Disorder" OR "Social Phobias" OR "phobia" OR "phobias" OR "posttraumatic stress disorder" OR "PTSD" OR "post-traumatic stress disorder" OR "post-traumatic stress disorders" OR "Posttraumatic Stress Disorders" OR "Posttraumatic Stress Disorder" OR "Posttraumatic Neuroses" OR "Posttraumatic Neurosis" OR "Post-Traumatic Neuroses" OR "Post-Traumatic Neurosis" OR "Traumatic Stress Disorder" OR "Traumatic Stress Disorders" OR "Battered Child Syndrome" OR "Combat Disorders" OR "Psychological Trauma" OR "Combat Disorder" OR "psychotrauma" OR psychotrauma* OR "Psychological Traumas" OR "generalized anxiety

disorder " OR "generalized anxiety disorder" OR "generalized anxiety disorders" OR "GAD" OR "panic" OR "Anxiety" OR anxiet*)) OR KW(("Art Therapy" OR "Color Therapy" OR "art therapy" OR "arts therapy" OR "art therapies" OR "arts therapies" OR "visual art therapy" OR "visual arts therapy" OR "Color Therapy" OR "Color Therapies" OR "Colour Therapy" OR "Colour Therapies" OR "painting therapy" OR "drawing therapy" OR "sculpting therapy" OR "clay therapy" OR "clay sculpting" OR clay model*) AND ("Anxiety Disorder" OR "anxiety disorder" OR "anxiety disorders" OR "Anxiety Neuroses" OR "Anxiety Neurosis" OR "Neurotic Anxiety State" OR "Neurotic Anxiety States" OR "Agoraphobia" OR "Separation Anxiety" OR "Neurocirculatory Asthenia" OR "Neurotic Disorders" OR "Obsessive-Compulsive Disorder" OR "Hoarding Disorder" OR "Panic Disorder" OR "Phobic Disorders" OR "Social Phobia" OR "Neurotic Disorder" OR "Obsessive-Compulsive Disorders" OR "OCD" OR "Hoarding Disorders" OR "Panic Disorders" OR "Phobic Disorder" OR "Social Phobias" OR "phobia" OR "phobias" OR "posttraumatic stress disorder" OR "PTSD" OR "post-traumatic stress disorder" OR "post-traumatic stress disorders" OR "Posttraumatic Stress Disorders" OR "Posttraumatic Stress Disorder" OR "Posttraumatic Neuroses" OR "Posttraumatic Neurosis" OR "Post-Traumatic Neuroses" OR "Post-Traumatic Neurosis" OR "Traumatic Stress Disorder" OR "Traumatic Stress Disorders" OR "Battered Child Syndrome" OR "Combat Disorders" OR "Psychological Trauma" OR "Combat Disorder" OR "psychotrauma" OR psychotrauma* OR "Psychological Traumas" OR "generalized anxiety disorder " OR "generalized anxiety disorder" OR "generalized anxiety disorders" OR "GAD" OR "panic" OR "Anxiety" OR anxiet*))
