# Supplementary material for: The effectiveness of art therapy for anxiety in adults: A systematic review of randomised and non-randomised controlled trials
Source: PLoS One. 2018 Dec 17;13(12):e0208716. doi: 10.1371/journal.pone.0208716 (PMC6296656; doi:10.1371/journal.pone.0208716)
Supplement: S1 Table — (PDF) [file pone.0208716.s003.pdf]

Risk of Bias

Authors	Title	Journal	Year	Protocol registration	Declaration of interest	Funding sources	Selection bias	Performance bias	Detection bias	Attrition bias	Reporting bias

Characteristics (1)

Authors	Title	Journal	Year	Aim of the study	Study type (RCT/nRCT)	Study population (based on inclusion/exclusion criteria, total number of participants)	Number of treated subjects	Number of controlled subjects

Characteristics (2)

Art therapy description	Duration, frequency, type (group or individual)	Individualized or protocolled treatment	Co-interventions	Control description	Outcome domains	Outcome measures (instruments)

Characteristics (3)

Results: anxiety symptoms	Results: quality of life	Results: psychotropic use	Results: comorbid depression (symptoms)	Perceived workings mechanism	Method of data analysis (statistics)	Authors' conclusions /summary	Reviewer opinion
