# Supplementary material for: The effectiveness of art therapy for anxiety in adults: A systematic review of randomised and non-randomised controlled trials
Source: PLoS One. 2018 Dec 17;13(12):e0208716. doi: 10.1371/journal.pone.0208716 (PMC6296656; doi:10.1371/journal.pone.0208716)
Supplement: S2 Table — (PDF) [file pone.0208716.s004.pdf]

Excluded studies with reasons for exclusion

Authors	Title	Journal	Published	Reason for exlcusion
Allen KN, Wozniak, DF	The language of healing: Women's voices in healing and recovering from domestic violence	Social Work in Mental Health	2011	no control group
Asawa P, Pateracki E	Reducing anxiety to technology: Utilizing expressive experiential interventions	Dissertation Abstracts International: Section B: The Sciences and Engineering	2004	dissertation, no peer reviewd publication
Campbell M, Decker K, Kruk K, Deaver, S	Art therapy and cognitive processing therapy for combat-related PTSD: A randomized controlled trial	Art Therapy	2016	only PTSD (no anxiety symptoms measured)
Curry NA, Kasser T	Can coloring mandalas reduce anxiety?	Art Therapy	2005	anxiety induction
de Morais AAH, Dalecio MAN, Vizmann A, Bueno,VLRD, Roecker S, Salvagioni, DAJ, Eler GJ	Effect on scores of depression and anxiety in psychiatric patients after clay work in a day hospital	Arts in Psychotherapy	2014	not only anxiety; no pre test
Eaton J, Tieber C	The effects of coloring on anxiety, mood, and perseverance	Art Therapy	2017	healthy population
Heynen E, Roest J, Willemars G, van Hooren S	Therapeutic alliance is a factor of change in arts therapies and psychomotor therapy with adults who have mental health problems	Arts in Psychotherapy	2017	no control group
Kim SK	A randomized, controlled study of the effects of art therapy on older Korean-Americans' healthy aging	Arts in Psychotherapy	2013	elderly
Kimport E, Hartzell E	Clay and anxiety reduction: A one-group, pretest/posttest design with patients on a psychiatric unit	Art Therapy	2015	no control group
Lande RG, Banks, Williams L., Francis JL, Gragnani C, Morin ML	Characteristics and effectiveness of an intensive military outpatient treatment program for PTSD	Journal of Aggression, Maltreatment and Trauma	2011	no control group
Selders M, Visser R, van Rooij W, Delfstra G, Koelen JA	The development of a brief group intervention (Dynamic Interpersonal Therapy) for patients with medically unexplained somatic symptoms: a pilot study	Psychoanalytic Psychotherapy	2015	AT was small part of large multidiciplinary treatment, no effect measures of AT
Swami V	Sketching people: Prospective investigations of the impact of life drawing on body image	Body Image	2017	no primary anxiety (negative body image)
Toroghi LM	The Effectiveness of Art Therapy On Housewives General Health, Anxiety and Self-efficacy in Tehran	European Psychiatry	2015	poster, no peer reviewed publication

Excluded studies with reasons for exclusion – from reference lists of systematic reviews

Authors	Title	Journal	Published	Reason for exlcusion
Andersson G, Paxling B, Roch-Norlund P et al.	Internet-based psychodynamic versus cognitive behavioral guided self-help for generalized anxiety disorder: a randomized controlled trial.	Psychosom 2012;81:344-55.	2012	no art therapy
Bell, C.E., & Robbins, S.J.	Effect of art production on negative mood: A randomized, controlled trial.	Art Therapy, 24(2), 71-75.	2007	induced worry in healthy population
Droždek B., Bolwerk N. (2010a).	Evaluation of Group Therapy With Traumatized Asylum Seekers and Refugees—The Den Bosch Model.	Traumatology, 16, 117–127. doi:10.1177/1534765610388298	2010	adults up to 70 years (>65). PTSD, and art therapy is part of a multidciplinary treatment and not seperatly tested.
Droždek B., Bolwerk N., Tol W. A., Kleber R. J. (2012).	Group therapy with male asylum seekers and refugees with PTSD: A controlled comparison cohort study of three day-treatment programs.	Journal of Nervous and Mental Disease, 200, 758–765.	2012	adults up to 70 years (>65). PTSD, and art therapy is part of a multidciplinary treatment and not seperatly tested.
Egger N, Konnopka A, Beutel ME et al.	Short-term cost-effectiveness of psychodynamic therapy and cognitive-behavioral therapy in social anxiety disorder: results from the SOPHO-NET trial.	J Affect Disord2015;180:21-8.	2015	no art therapy
Knijnik DZ, Salum GA, Jr., Blanco C et al.	Defense style changes with the addition of psychodynamic group therapy to clonazepam in social anxiety disorder.	J Nerv Ment Dis 2009;197:547-51.	2009	no art therapy
Leichsenring F, Salzer S, Beutel ME et al.	Psychodynamic therapy and cognitive-behavioral therapy in social anxiety disorder: a multicenter randomized controlled trial.	Am J Psychiatry 2013;170:759-67.	2013	no art therapy
Leichsenring F, Salzer S, Beutel ME et al.	Long-term outcome of psychodynamic therapy and cognitive-behavioral therapy in social anxiety disorder.	Am J Psychiatry 2014;171:1074-82.	2014	no art therapy
Leichsenring F, Salzer S, Jaeger U et al.	Short-term psychodynamic psychotherapy and cognitive-behavioral therapy in generalized anxiety disorder: a randomized, controlled trial.	Am J Psychiatry2009;166:875-81.	2009	no art therapy
