# Supplementary material for: The effectiveness of art therapy for anxiety in adults: A systematic review of randomised and non-randomised controlled trials
Source: PLoS One. 2018 Dec 17;13(12):e0208716. doi: 10.1371/journal.pone.0208716 (PMC6296656; doi:10.1371/journal.pone.0208716)
Supplement: S3 Table — (PDF) [file pone.0208716.s005.pdf]

Risk of Bias

Authors	Title	Journal	Year	Protocol registration	Declaration of interest	Funding sources	Selection bias	Performance bias	Detection bias	Attrition bias	Reporting bias
Henderson P, Rosen D, Mascaro N	Empirical study on the healing nature of mandalas	Psychology of Aesthetics, Creativity, and the Arts	2007	Approval for study from the University's review board.	The authors not specifically focus on conflict of interest.	Not mentioned	Unclear: randomization method is not described, as well as the allocation concealment. Included participants were randomized over experimental and control groups. It is unclear how gender and type of trauma is distributed.	Unclear: blinding is not possible. True nature of the study was not told to participants. Debriefing immediately following the intervention was incomplete to protect validity of follow up measurements.	Unclear: Self report questionnaires were used to prevent detection bias. Final interviews of participants of the experimental group cannot be blinded.	Unclear: It is not specifically mentioned whether all data sets were complete.	Low: Data set seems to be complete. No indications for selective reporting.
Sandmire D, Gorham S, Rankin N, Grimm D	The influence of art making on anxiety: A pilot study	Art Therapy	2012	Not mentioned	unknown	Not mentioned.	Unclear: randomization method is not described, as well as the allocation concealment. Randomization resulted in two comparable groups (in terms of measures used).	High: Blinding was not possible, and control group did not receive treatment.	Unclear: Self report questionnaires were used. It is not clear whether blinding of outcome assessors was applied.	Low: Only 2 pre-activity data were missing, and reported. 2 students of experimental group did not answer the trait questions of the STAI prior to the art making.	Low: No indications for selective reporting.
Yu Y, Yu Ming C, Yue M, Hai Li J, Ling L ,	House-Tree-Person drawing therapy as an intervention for prisoners' prerelease anxiety	Social Behavior and Personality	2016	Not mentioned	unclear / not addressed	Not mentioned.	Unclear: randomization method is not described, as well as the allocation concealment. Resulted in two comparable groups (in terms of measures used). Unclear whether demographic parameters and psychopathology of control and experimental groups are equal.	Unclear: blinding is not possible. If this influenced the outcomes cannot be judged.	Unclear: Self report questionnaires were used, but the circumstances (privacy) under which measures are used are not clear.	Low: incomplete data files were discarded from analysis.	Low: no indications for selective reporting.

Characteristics (1)

Authors	Title	Journal	Year	Aim of the study	Study type (OBS/CT/RCT)	Study population (based on inclusion/exclusion criteria, total number of participants)	Number of treated subjects	Number of controlled subjects
Henderson P, Rosen D, Mascaro N	Empirical study on the healing nature of mandalas	Psychology of Aesthetics, Creativity, and the Arts	2007	Examine the healing aspects of creating mandalas on mental health.	RCT	Undergraduate students experiencing one or more traumatic stressors and at least moderate levels of PTSD symptom severity. Exclusion occurred when potential participants were also in psychotherapy or taking psychotropic medication.	19	17
Sandmire D, Gorham S, Rankin N, Grimm D	The influence of art making on anxiety: A pilot study	Art Therapy	2012	To assess if art-making leads to (significant) anxiety reduction, compared to a control group; Study the psychological effects of art making with the emphasis on the influence on anxiety	RCT	Undergraduate first year students (N=57; 45 female, 12 male), in a stressful time: a week prior to final exams. Students attending liberal arts college in US, with no anxiety disorder and no use of medication known to influence the central nervous system (e.g. for depression or ADHD). 57 first-year undergraduate student of different ethnicity attending a small liberal arts college in USA one week prior to final exams for the semester. Exclusion of students having any anxiety disorder or other documented mental illness or using medication known to influence the central nervous system.	29	28
Yu Y, Yu Ming C, Yue M, Hai Li J, Ling L ,	House-Tree-Person drawing therapy as an intervention for prisoners' prerelease anxiety	Social Behavior and Personality	2016	Examining the feasibility of using HTP drawing therapy as an intervention to reduce prisoners'prelease anxiety and assessing its effectiveness. Examine the use of the HTP drawing to reduces prioners' prelease anxiety	RCT (quasi-experimental)	72 male prisoners with sentences of 2 to 6 months still to serve before release with a HAM-A score of 14 or more and a SAS score of 50 or more at the start of the intervention.	36	36

Characteristics (2)

Author	Year	Art therapy description	Duration, frequency, type (group or individual)	Individualized or protocolled treatment?	Co-interventions	Control description	Outcome domains	Outcome measures (instruments)
Henderson P, Rosen D, Mascaro N	2007	Mandala creation (represent feelings or emotions related to personal trauma within the contour of a circle), concluded by an interview about the meaning of symbols at follow up.	3 times on 3 consecutive days, 20 minutes per session.	Groups of 5-10 individuals; protocolled treatment.	None	Three specific drawing assignments of 20 minutes each.	Mental health problems, spirituality and Limbic Languidness.	PDS=Post traumatic Disorder Scale (self report measure); the BDI-II=Beck Depression Inventory (second self report version); the STAI=State-Trait Anxiety Inventory (self report measure); the SMS=Spriritual Meaning Scale (self report measure) and the PILL=Pennebaker Inventory of Limbic Languidness (self report measure?).
Sandmire D, Gorham S, Rankin N, Grimm D	2012	One choice out of five art-making activities: mandala design (colouring a pre-designed mandala), painting free form, collage making, clay (a pleasing form), drawing (still life objects); social interaction was allowed, no use of electronic devices	30 minutes, once. Group and social interaction within groups was allowed..	Protocolled, but free choice out of five art-making activities	None	Sitting in comfortable chairs, social interaction was allowed, no use of electronic devices	Anxiety symptoms: state anxiety and trait anxiety	STAI
Yu Y, Yu Ming C, Yue M, Hai Li J, Ling L ,	2016	House-Tree-Person test: drawing a house, a tree and a person, as well as some other objects on a sheet of paper. The house is regarded as the projection of family, the tree represents the environment and the person represents self-identification. Protocol: 2B-pencil, A4-sheet of paper and a eraser. No set time to complete their drawing.	10 times over a period of 5 weeks; 30+40-60 minutes, twice a week, 5 weeks, small groups of prisoners from different cells	protocolled	Group interview (40-60min) twice a week over a period of 5 weeks	Only group interview (40-60min) twice a week over a period of 5 weeks	Anxiety	HAM-A and SAS

Characteristics (3)

Author	Year	Results: anxiety symptoms	Results: quality of life	Results: psychtropic use	Results: comorbid depression (symp)	Percieved workingsmechanism	Method of data analysis (statistics)	Authors' conclusions /summary	Reviewer opinion
Henderson	2007	Pre /post /follow up (anxiety):<br>Mandala group: 45.05 (SD=10.75) / 41.16 (SD=11.30) / 40.95 (SD=11.54<br>Control group: 49.05 (SD=12.29) / 44.05 (SD=10.12) / 42.0 (SD=13.26)<br><br>Pre / post / follow up (PDS):<br>Mandala group: 19.37 (SD=7.06) / 18.05 (SD=9.71) / 13.42 (SD=8,45)<br>Control group: 15.71 (SD=6.89) / 15.41 (SD=8.49) / 15.47 (SD=8.62)	not studied	not studied	Decline from 17.95, SD=9.26 to 16.63, SD=11.74 in mandala group versus a decline from 15.35, SD=7.11 to 13.35, SD=7.03 in the control group. Outcomes at follow-up: further decline to 13.95, SD=9.50 in mandala group versus 13.06, SD=8.55 in control group.	The significantly lowered levels of traumatic symptoms in the experimental group at follow up may suggest that mandala creation leads to changes deep within cognition.	One way analyses of covariance (ANCOVA)	Mandala creation leads to a decrease of PTSD symptom severity (at follow up).	The authors' conclusion may be influenced by factors not accounted for.
Sandmire	2012	Mean Pre-activity / Post-activity State Control Group: 36.2±8.8 / 36.0±10.9; t=0.08. Mean Pre-activity / Post activity State Experimental Group: 39.3±9.4 / 29.5±8.6; t=3.98 (p<0.01).<br>Mean Pre-activity / Post-activity Trait Control Group: 38.2±10.2 / 37.3±11.2 (t=1.19). Mean Pre-activity / Post activity Trait Experimental Group: 39.1±5.8 / 33.3±6.1 (t=4.20 (p<0.01).<br>There was no contribution of type of art-making activity or subjects'sex to the outcomes.<br>Decline state anxiety scores experimental group from 39.3 (SD=9.4) to 29.5 (SD=8.6)** versus decline from 36.2 (SD=8.8) to 36.0 (SD=10.9) in control group. Type of art-making not significantly important.	not studied	not studied	not studied	Art therapy offers a bottom-up approach to anxiety, in a non-verbal, tactile and visual manner (Sarid & Huss, 2010). A trance-like state (in flow) occurs during art-making (Csikszentmihalyi, 1997), due to the tactile and visual experience as well as the repetitive muscular activity inherent in art making. Entering a flow-like state can decrease anxiety.	Two-tailed paired and unpaired Students t-tests: within and between groups. A two-by-two factorial ANOVA was used to determine whether the type of activity or sex influenced the pre- to post-activity STAI scores.	30 minutes of art making significantly reduces participants' state-related anxiety (as measured by the STAI). The relaxing effect of art making is significant. Entering a flow-like state can decrease anxiety.	Liberal arts students are likely to have positive feelings about art-making and might therefor experience more decrease in anxiety then students from another discipline, and/or with less art-making experience.

Yu	2016	HAM-A experimental group: 24.36(SD 9.11) / 17.42 (SD 10.42); t=5.295; df=32; p 0.001. HAM-A control group: 24.75 (SD 6.14) / 25.22 (SD 7.37); t=--0.457; df=35, p=.650<br>SAS experimental group: 62.63 (SD 9.46); t=3.065; df=32; p 0.004. SAS control group: 62.57 (SD 7.36) / 66.11 (SD 10.41); t=-2.214; df=35; p 0.033; Decline of HAM-A scores in experimental group from 24.36(SD= 9.11) to 17.42 (SD =0.42)** versus an increase from 24.75 (SD=6.14) to 25.22 (SD=7.37) in control group. Decline of SAS scores in experimental group from 62.63 (SD=9.46) to 56.78 (SD=11.64)** versus an increase from 62.57 (SD=7.36) to 66.11 (SD=10.41) in control group.	not studied	not studied	not studied	The content of the art making and reflection upon gives insights in (wrong) cognitions that can be counselled afterwards. HTP-drawing is a natural, easy mental intervention technique through which counsellors can guide prisoners to form helpful cognition and behaviours within a relative relaxing and well-protected psychological environment. The HTP-drawing sheds light on the prisoner's inner world. Feelings, emotions and impulses can be acknowledged and then symbolically embodied in drawings. Negative feelings can be transformed into a motivating force. With guidance of counsellors during the open interview stage of HTP therapy, prisoners are more likely to achieve a better mental state, forming the correct cognition, contributing to a healthy personality. It helps prisoners regain their sense of self-control.	bilateral paired samples t tests.	Anxiety scores are significantly reduced in the experimental group as the result of HTP drawings. HTP drawings also indicated reduced anxiety levels.<br>This shows the effectiveness of the HTP-drawing therapy in reducing prisoners' prelease anxiety	Anxiety scores are significantly reduced in the experimental group, which is possibly the result of HTP drawings and consecutive counselling, but the effectiveness cannot be proved since no between-group outcomes are presented.
